# Emotion-RGC net: A novel approach for emotion recognition in social media using RoBERTa and Graph Neural Networks

**DOI:** 10.1371/journal.pone.0318524

**Published:** 2025-03-03

**Authors:** Jiangting Yan, Pengju Pu, Liheng Jiang

**Affiliations:** 1 School of Art & Design, Shaanxi Fashion Engineering University, Xi’an, China; 2 CVS Health Corporation, Woonsocket, Rhode Island, USA; The University of Lahore, PAKISTAN

## Abstract

Emotion recognition in social media is a challenging task due to the complex and unstructured nature of user-generated content. In this paper, we propose Emotion-RGC Net, a novel deep learning model that integrates RoBERTa, Graph Neural Networks (GNN), and Conditional Random Fields (CRF) to enhance the accuracy and robustness of emotion classification. RoBERTa is employed for effective feature extraction from unstructured text, GNN captures the propagation and influence of emotions through user interactions, and CRF ensures global consistency in emotion label prediction. We evaluate the proposed model on two widely-used datasets, Sentiment140 and Emotion, demonstrating significant improvements over traditional machine learning models and other deep learning baselines in terms of accuracy, recall, F1-score, and AUC. Emotion-RGC Net achieves an accuracy of 89.70% on Sentiment140 and 88.50% on Emotion, highlighting its effectiveness in handling both coarse- and fine-grained emotion classification tasks. Despite its strong performance, we identify areas for future research, including reducing the model’s reliance on large labeled datasets, improving computational efficiency, and incorporating temporal dynamics to capture emotion evolution in social networks. Our results indicate that Emotion-RGC Net provides a robust solution for emotion recognition in diverse social media contexts.

## Introduction

With the widespread use of the internet, social media has become a crucial platform for daily communication and information sharing. Users express their emotions and opinions through various forms such as text, images, and videos, making emotion analysis a hot research topic in fields like social sciences, marketing, and intelligent human-computer interaction. Emotion recognition is not merely about determining sentiment polarity (e.g., positive or negative); more importantly, it involves identifying nuanced emotion categories (e.g., anger, happiness, sadness) to better understand users’ psychological states [1]. As a result, the challenge of efficiently and accurately identifying and classifying emotions from massive volumes of social media data has become a research area of significant practical value. In the commercial realm, emotion recognition can help companies better understand user feedback, optimize products and services, and improve customer satisfaction [2]. In social network analysis, emotion recognition aids in uncovering patterns of emotion propagation, potentially enabling the early detection of collective emotional shifts and the prevention of rumors and negative emotions from spreading. Therefore, developing an automated model capable of accurately capturing the emotions of social media users is not only vital for business applications but also holds broad social significance. However, social media text often includes unstructured and noisy data, such as slang, emoticons, and abbreviations, which complicates the task of emotion recognition [3,4]. Moreover, the relationships between users and the patterns of emotion propagation within networks are also complex factors that must be considered in emotion analysis. As such, existing emotion recognition methods face challenges in efficiently extracting emotional features from complex data and capturing the relationships driving emotion propagation.

In recent years, the rapid development of deep learning technologies has led to significant advances in emotion analysis. Methods based on convolutional neural networks (CNNs), recurrent neural networks (RNNs), and pre-trained language models (such as BERT and RoBERTa) have demonstrated powerful performance in natural language processing tasks. These models automatically extract features from data, reducing the need for manually designed features, which traditional emotion analysis methods heavily relied upon [5]. For instance, pre-trained models like BERT and RoBERTa, through large-scale unsupervised training, capture rich contextual information, enabling them to achieve outstanding performance in downstream tasks such as emotion classification. Despite the many advantages deep learning brings to emotion analysis—such as improving generalization and reducing reliance on labeled data—there are still several unresolved issues [6]. First, social media texts often contain informal language, emoticons, and ambiguity, making it difficult for text-based feature extraction methods alone to accurately capture users’ true emotions. Second, the propagation of emotions within social networks is a complex process, where emotions from different users often influence each other. Traditional emotion analysis methods generally overlook the relational structure between users, leading to suboptimal performance when dealing with large-scale emotion propagation in social networks [7]. Moreover, while pre-trained language models perform well in feature extraction, they typically lack the ability to model emotion propagation and user relationships, limiting their applicability in social media emotion analysis.

To address these issues, this paper proposes a novel emotion recognition method, Emotion-RGC Net, which combines the strengths of the RoBERTa pre-trained language model and Graph Neural Networks (GNNs) to enhance the accuracy of emotion recognition in social media. RoBERTa is leveraged to extract rich emotional features from textual data, capitalizing on its powerful capabilities in natural language understanding [8]. GNNs, on the other hand, excel at processing user relationship networks and can capture the interactions and propagation of emotions between users [9]. Additionally, Conditional Random Fields (CRF) are introduced in the post-processing phase to further refine the emotion classification results, considering the interdependencies between emotion categories [10]. This innovative multi-module model not only accurately identifies emotions in social media but also models the propagation of emotions, overcoming the limitations of traditional methods that fail to effectively utilize user relationship networks.

The main contributions of this paper are as follows:

- This paper propose an end-to-end emotion recognition model that combines RoBERTa and GNN, effectively capturing both textual and relational features.
- The paper enhance emotion classification accuracy by incorporating a Conditional Random Field (CRF) layer.
- This paper provide an in-depth analysis and modeling of emotion propagation mechanisms, expanding the application scenarios of emotion analysis.

## Related work

### Multimodal emotion analysis

In recent years, multimodal emotion analysis has gained widespread attention. Compared to unimodal approaches (such as pure text), multimodal emotion analysis combines different types of data sources (e.g., text, audio, video) to capture emotional expressions more comprehensively and accurately through the fusion of multiple information streams. For instance, in video-based emotion analysis, researchers do not only analyze textual data but also integrate non-verbal cues such as speech tone and facial expressions to build a multimodal understanding of emotions [11,12]. This multi-angle approach effectively compensates for the limitations of single-modality data and significantly enhances the accuracy and robustness of emotion recognition. The core of multimodal emotion analysis lies in feature fusion—how to effectively combine data from different modalities and process them in a unified way. This process typically relies on advances in deep learning, particularly methods like convolutional neural networks (CNNs) and recurrent neural networks (RNNs), which can automatically extract high-dimensional emotional features from raw audio, video, and textual data [13]. Recently, researchers have begun experimenting with more advanced models, such as Transformer models based on attention mechanisms, to strengthen the connections between different modalities and better capture emotional information. Additionally, by employing joint learning on multimodal data, models can further improve their performance in complex emotional scenarios, such as when users express mixed emotions [14]. The fusion of multiple modalities helps to accurately recognize these nuanced emotional states.

While multimodal emotion analysis offers significant advantages, it also faces considerable challenges. Data from different modalities often have varying formats and temporal characteristics, making data fusion complex. Text processing typically relies on the order of words or sentences, whereas audio and video require temporal information to capture emotional changes [15]. Synchronizing and coordinating information across modalities is one of the key difficulties in multimodal emotion analysis. Furthermore, multimodal approaches usually require substantial computational resources and access to high-quality multimodal datasets, which can be costly to acquire and process. Another issue is data inconsistency—there may be redundancy or conflicts between information from different modalities, and finding ways to preserve relevant information while reducing noise is an ongoing challenge.

Despite these challenges, the potential of multimodal emotion analysis to accurately recognize emotions remains enormous, especially in social media contexts where users often express emotions not only through text but also through images, videos, and even audio [16]. Multimodal emotion analysis thus provides a more comprehensive perspective for emotion recognition. With advancements in deep learning technologies and hardware infrastructure, future research in this field is expected to overcome current challenges, allowing multimodal emotion analysis to play a role in a wider range of applications [17]. In this context, combining textual data with other modalities for emotion analysis is becoming a trend, but in the practical application of social media, text remains the primary medium for conveying information. Efficiently utilizing deep learning techniques for unimodal text-based emotion analysis remains a key research direction. This paper adopts this perspective and proposes a text-based emotion recognition model, Emotion-RGC Net.

### Social media emotion propagation network models

On social media platforms, emotions are expressed not only through individual posts and interactions but also propagate through exchanges, reposts, and comments between users. This process of emotion propagation makes emotion analysis not just an individual-level task but a networked phenomenon [18]. In social networks, emotions often spread and evolve in complex ways between users. A user’s emotional state can directly influence other closely connected users, creating a "contagion effect." For instance, when a user expresses strong emotions in a social media post, it can trigger responses or reposts from others, further amplifying and spreading the emotion across a larger network [19]. Research shows that this kind of emotional propagation in social networks follows certain patterns, particularly in how different network structures can affect the strength and speed of emotion diffusion. Effectively modeling these emotional relationships has become a key challenge in improving the accuracy of emotion recognition [20]. To capture and model emotion propagation patterns on social media, Graph Neural Networks (GNNs) have gained significant attention in recent years. GNNs are capable of processing graph-structured data, where nodes represent users and edges represent interactions between them, allowing the construction of a network model that can simulate emotion propagation. Unlike traditional emotion analysis models, GNNs iteratively update the representations of nodes, learning the emotional interaction patterns between users [21]. For example, a user’s emotional state is influenced not only by their own textual features but also by the emotional states of connected users. Through this process, GNNs can capture the complexity of emotion propagation in social networks, providing richer information for emotion classification.

Recent advancements in emotion propagation network models based on GNNs have yielded promising results. Studies have utilized GNNs to analyze interaction patterns between users and found that emotion propagation follows different paths and intensities depending on the structure of the social network [22]. On platforms like Twitter, emotional interactions between users often involve multiple forms of engagement, such as retweets, comments, and likes. By modeling these interactions with GNNs, it becomes possible to predict users’ emotional states more accurately and even forecast the spread of emotions in advance. However, GNNs still face challenges in modeling emotion propagation [23]. The relationships between users are highly complex, and effectively modeling emotion propagation at scale remains a significant issue. Additionally, emotional propagation is not solely dependent on user relationships but is also influenced by other factors, such as topic trends, temporal dynamics, and external events. Solely relying on graph-based models is insufficient to address all challenges in emotion analysis [24]. Incorporating these dynamic factors into graph models to improve the accuracy of emotion propagation prediction is an important direction for future research. The proposed Emotion-RGC Net aims to address these challenges by combining the strengths of RoBERTa and GNN. It leverages the emotional information from textual data while accounting for the relational structures between users, thereby enabling more efficient and accurate emotion recognition on social media.

### Application of pre-trained language models

With the rapid advancements in deep learning within the field of Natural Language Processing (NLP), pre-trained language models have become one of the mainstream methods for emotion analysis. These models are pre-trained on large-scale, unsupervised corpora, learning rich contextual information and effectively transferring to downstream tasks such as sentiment recognition and emotion classification [25]. This "pre-training and fine-tuning" paradigm significantly enhances the performance of emotion analysis, especially in tasks where the amount of data is limited or the cost of annotation is high. The successful application of pre-trained models like BERT and RoBERTa has notably improved the accuracy and robustness of textual emotion recognition. In recent years, the application of BERT (Bidirectional Encoder Representations from Transformers) in emotion analysis tasks has garnered significant attention [26]. By utilizing a bidirectional Transformer structure, BERT can simultaneously capture both the preceding and succeeding context of a sentence, leading to a deeper understanding of its semantics and emotional content. Building upon BERT, further improved versions have been developed, such as RoBERTa (Robustly Optimized BERT Approach), which enhances language representation by increasing the amount of pre-training data, extending training time, and optimizing hyperparameters [27]. These pre-trained models can capture not only semantic information at the word and phrase levels but also implicit emotional signals within the context, thus providing a clear advantage in emotion classification tasks.

The application of pre-trained language models in emotion analysis brings several distinct advantages. By leveraging unsupervised learning on large-scale text corpora, these models significantly reduce the reliance on labeled data for emotion analysis tasks. This is particularly critical in scenarios where emotion-labeled datasets are scarce, such as in social media, where obtaining high-quality, emotion-annotated datasets is often challenging [28]. Pre-trained models enable knowledge transfer from the pre-training phase to emotion analysis tasks, thereby enhancing model performance. Moreover, these models exhibit strong generalization capabilities, allowing them to easily adapt to various emotion classification tasks, whether based on broad emotional categories (e.g., anger, joy, sadness) or more fine-grained emotion distinctions [29]. Pre-trained language models also tend to generalize well, not only performing exceptionally on training data but also handling the diversity and complexity of language in real-world applications, thus avoiding issues like overfitting.

Despite the tremendous success of pre-trained models in emotion analysis tasks, several limitations remain. Pre-trained models primarily focus on textual features, while in social media emotion analysis, the relationships and interactions between users are equally critical. While these models can extract emotional features from individual texts, they often overlook the propagation and evolution of emotions within a user network, limiting their potential for complex social network-based emotion analysis [30]. Additionally, large-scale models like RoBERTa require significant computational resources during both training and inference, which poses challenges when applying these models to large-scale social media data [8]. To overcome these limitations, this paper not only leverages RoBERTa to extract emotional features from text but also incorporates Graph Neural Networks (GNNs) to capture the emotional propagation relationships between users. This combination effectively addresses the shortcomings of relying solely on pre-trained models, enabling Emotion-RGC Net to consider both textual and network structure features in social media emotion recognition, thus improving the accuracy and robustness of emotion classification.

## Methods

In the field of credit risk assessment, despite the demonstrated potential of deep learning models such as RNNs, LSTM networks, and GRUs in processing sequential data, these methodologies exhibit significant limitations in capturing long-term dependencies. Addressing these challenges, we propose a sophisticated hybrid model that synthesizes the strengths of LSTM and GRU architectures to enhance the accuracy and computational efficiency in credit risk prediction. Our model aims to refine the existing credit scoring paradigms by equilibrating computational efficiency with the proficiency to capture intricate temporal patterns. The subsequent section delineates this model in comprehensive detail.

### Overall model: emotion-RGC net

**Fig 1:**
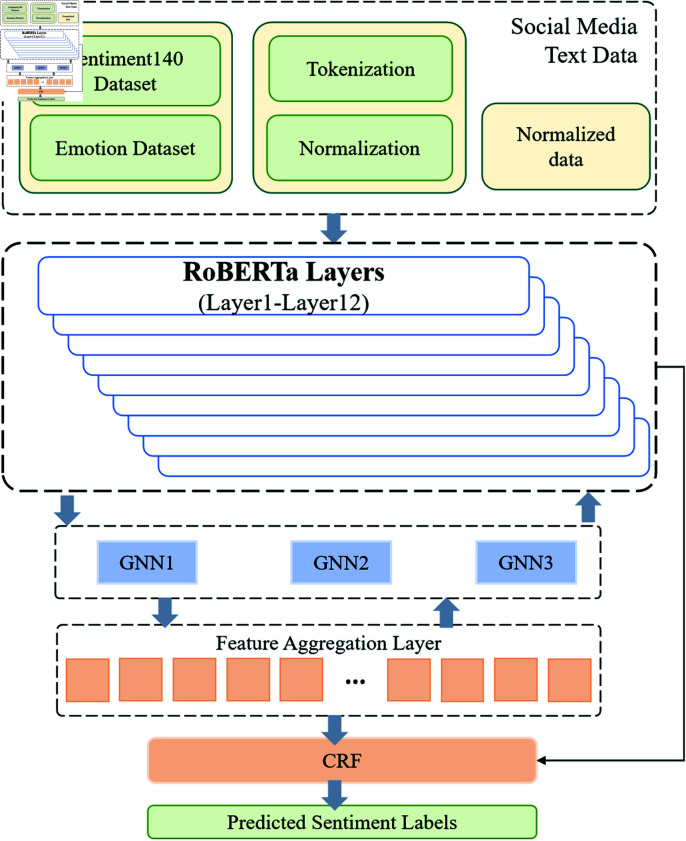
The architecture of Emotion-RGC Net. The RoBERTa module extracts emotion features from social media text; the GNN module builds and updates the social relationship network between users, capturing emotion propagation patterns; the CRF module globally optimizes the final emotion classification to ensure coherence and consistency in emotional states.

In this paper, we propose Emotion-RGC Net, a multi-module deep learning model designed for emotion recognition on social media. The innovation of this model lies in its ability to address multi-layered challenges in emotion analysis by combining a pre-trained language model (RoBERTa), a Graph Neural Network (GNN), and Conditional Random Fields (CRF). The overall architecture of the model is shown in Fig 1, consisting of three core modules: RoBERTa for emotion feature extraction from text, GNN for modeling user relationships and capturing emotion propagation, and CRF for post-processing and optimizing emotion classification.

In the text input processing phase, the model receives user-generated text data from social media as input. Given that social media text is often highly unstructured, containing emoticons, non-standard language, and more, Emotion-RGC Net employs the RoBERTa model for deep feature extraction. RoBERTa, with its bidirectional Transformer architecture, captures fine-grained emotional cues and language dependencies from the context. After preprocessing, the text is embedded into vector representations, forming feature vectors rich in semantic and emotional information.

Next comes the user relationship modeling and emotion propagation phase. Emotion-RGC Net utilizes GNN (Graph Neural Network) to capture the network of relationships between social media users. GNN treats users as nodes in the graph and interactions (such as comments, shares, or likes) as edges, constructing a social network structure among users. The text features generated by RoBERTa are passed into the GNN model as initial node (user) representations. Through a multi-layer message-passing mechanism, each user node aggregates emotional information from its neighboring nodes, capturing the emotion propagation patterns between users. After several iterations, the GNN updates each node’s emotion representation, ultimately producing feature vectors that reflect the propagation characteristics of emotions across the social network.

In the third stage, the model performs emotion classification and optimization. The emotion features processed by GNN are input into CRF (Conditional Random Fields), which further optimizes the emotion classification globally. CRF models the dependencies between emotional states, ensuring that the classification results remain consistent across sequences of social interactions. For example, in an interaction chain, users’ emotions often correlate with one another, and CRF captures this dynamic, avoiding disjointed predictions. Additionally, CRF leverages the contextual emotional information provided by the GNN to further enhance the accuracy and coherence of the emotion classification.

As shown in Fig 1, RoBERTa as an emotional feature extraction module, it is responsible for extracting the vector representation with emotion information from the input text. These emotional characteristics usually contain rich emotional information, including the emotional polarity (positive, negative, etc.) and the intensity of the emotion. These features will be sent to the graph neural network (GNN) for further processing and aggregation. The role of GNN in this model is to spread emotional features through the graph structure information between nodes, simulating the propagation process of emotion in social networks. In each layer of GNN, the emotional features are updated according to the emotional information of the adjacent nodes, eventually forming a global understanding of the overall emotional transmission. The CRF module plays a role in optimizing the emotional transmission path. During emotion transmission, inconsistency or noise of emotion information may occur, and the CRF helps the model to select the most consistent emotion sequence and optimizes it in a way of maximizing the global conditional probability. By calculating the transition probability between labels and the relationship between observed features and labels, CRF provides an effective global optimization strategy for the model, which further improves the accuracy and consistency of emotion transmission.

These modules work closely together, and Emotion-RGC Net can model the transmission process of emotion more accurately, and effectively process the local and global dependence of emotional information. RoBERTa The extracted emotion features provide powerful local information for GNN, while the graph structure aggregation ability of GNN provides the optimization basis for CRF, finally forming a powerful and efficient model of emotion transmission. This modular design not only improves the expressive power of the model, but also enhances its robustness in complex emotional transmission scenarios.

The running process of the Emotion-RGC Net model is shown in Algorithm 1.

**Algorithm 1: Training Process of Emotion-RGC Net**
  **Input:** Sentiment140 Dataset, Emotion Dataset
  **Output:** Trained Emotion-RGC Net, Evaluation Metrics
  **Function** *TrainEmotionRGCNet(**Sentiment*140 , *Emotion**)***:**
    **Initialize** *Model* ← *Emotion* − *RGCNet* ;
    **Initialize** *Optimizer* ← *Adam* ( *α* = 0 . 001 )  ;
    **Initialize** *LossFunction* ← *CrossEntropyLoss* ;
    **for** **epoch* ← 1 to *max*_*epochs** **do**
      **For each batch** *B* ∈ { *Sentiment*140 , *Emotion* }  { *inputs* , *labels* ← *B*;
      *outputs* ← *Model* ( *inputs* ) ;
      **Calculate loss:** *Loss* ← *LossFunction* ( *outputs* , *labels* ) ;
      **Backpropagate loss and update model parameters:** *Optimizer* . *update* ( *Loss* ) ;
      }
        **if** **epochmodeval*_*interval* = 0* **then**
          *predictions* ← *Model* ( *test*_*inputs* ) ;
          *accuracy* ← *Accuracy* ( *predictions* , *test*_*labels* ) ;
          *recall* ← *Recall* ( *predictions* , *test*_*labels* ) ;
          *f*1 ← *F*1_*score* ( *predictions* , *test*_*labels* ) ;
          *auc* ← *AUC* ( *predictions* , *test*_*labels* ) ;
          **if** **accuracy* > *best*_*accuracy** **then**
            *best*_*accuracy* ← *accuracy*;
            *best*_*model* ← *Model*;
          **end**
        **end**
      **end**
      **Return** *best*_*model* , *best*_*accuracy* , *recall* , *f*1 , *auc*

### Emotion feature extraction module: RoBERTa

In the Emotion-RGC Net model, RoBERTa serves as the key module for emotion feature extraction. Using a bidirectional Transformer structure, it extracts emotional cues from text and generates high-dimensional feature vectors suitable for subsequent processing. The strength of the RoBERTa model lies in its deep language representations learned from extensive pre-training on large-scale corpora, showcasing exceptional capability when handling social media texts with complex semantic dependencies [8,31].

**Fig 2:**
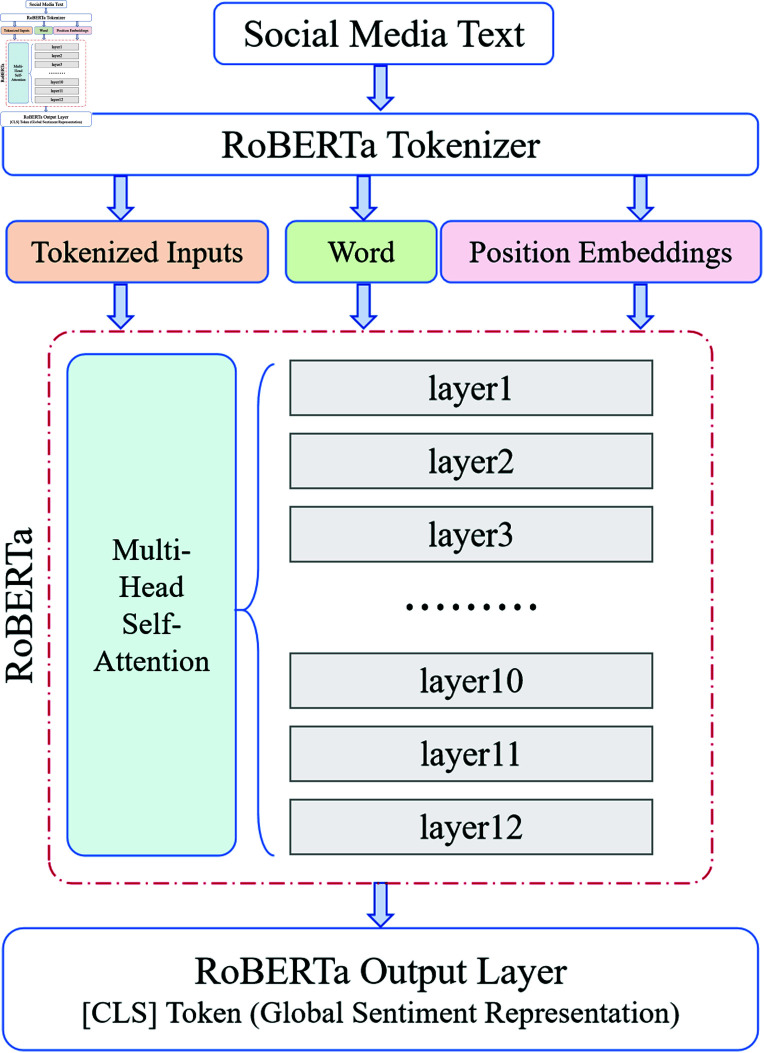
Structure of the RoBERTa module. Input text is first processed through word and positional embeddings to generate context-aware vector representations. After being processed by the multi-head self-attention mechanism, the model gradually produces emotion features containing global contextual information, with the final global emotion representation output through the [CLS] token.

As shown in Fig 2, RoBERTa is based on the BERT architecture and consists of multiple layers of bidirectional Transformer encoders. Transformers allow the model to attend to both preceding and following context simultaneously within a sentence. Compared to traditional unidirectional models, RoBERTa’s bidirectional approach enables it to more comprehensively capture both the semantic and emotional information present in the text. Each Transformer layer comprises two sub-layers: a self-attention mechanism and a feed-forward neural network.

In the Emotion-RGC Net model, RoBERTa is responsible for extracting emotion features from social media text. The process includes input preprocessing, generating contextual feature representations through the Transformer layers, and outputting the final emotion feature vector.

The input text is first tokenized, mapping the text sequence $T=[t_{1},t_{2},\ldots ,t_{n}]$ into a series of subword units (tokens) and representing them as embedding vectors. Suppose the tokenized input sequence is $X=[x_{1},x_{2},\ldots ,x_{n}]$, then the embedding for each token $x_{i}$ is defined as:


$$\begin{array}{c} E_{i}=W_{e}(x_{i})+W_{p}(i) \end{array}$$ (1)

where $W_{e}(x_{i})$ is the word embedding from the matrix $W_{e}\in {\mathbb{R}}^{|V|\times d}$, and $W_{p}(i)$ is the positional embedding from matrix $W_{p}\in {\mathbb{R}}^{n\times d}$, where *d* is the dimension of the embedding vector, *V* is the vocabulary size, and *n* is the input sequence length. Through word and positional embeddings, the text sequence is transformed into context-aware representations, which serve as input to the Transformer layers.

Each layer of the Transformer contains a multi-head self-attention mechanism to capture the contextual dependencies within the input text sequence. For each input sequence $X=[x_{1},x_{2},\ldots ,x_{n}]$, the self-attention mechanism computes attention scores that capture the relationships between each token and other tokens in the sequence:


$$\begin{array}{c} \text{Attention}(Q,K,V)=\text{softmax}\left(\frac{QK^{T}}{\sqrt{d_{k}}}\right)V \end{array}$$ (2)

where $Q=XW_{Q}$, $K=XW_{K}$, and $V=XW_{V}$, with $W_{Q},W_{K},W_{V}\in {\mathbb{R}}^{d\times d_{k}}$ as the projection matrices for queries, keys, and values, and $d_{k}$ being the dimension of each head. For multi-head attention, the formula expands to:


$$\begin{array}{c} \text{MultiHead}(Q,K,V)=\text{Concat}({\text{head}}_{1},\ldots ,{\text{head}}_{h})W_{O} \end{array}$$ (3)

where ${\text{head}}_{i}=\text{Attention}(Q_{i},K_{i},V_{i})$, and $W_{O}\in {\mathbb{R}}^{hd_{k}\times d}$ is the output weight matrix, with *h* as the number of attention heads.

This mechanism allows each token to attend to different features of other tokens across multiple attention heads, enhancing the model’s ability to understand complex texts, particularly in capturing long-range dependencies. After being processed through multiple Transformer layers, the contextual feature representation generated is:


$$\begin{array}{c} H_{l}=\text{LayerNorm}(H_{l-1}+\text{MultiHead}(H_{l-1})) \end{array}$$ (4)

where $H_{l}\in {\mathbb{R}}^{n\times d}$ is the output matrix of the *l*-th layer, representing the contextual feature representation after multi-head attention and residual connection.

After several Transformer layers, RoBERTa produces the contextual feature representation matrix $H_{L}\in {\mathbb{R}}^{n\times d}$, where *L* represents the output of the final layer. To generate a global representation of the text, the special [CLS] token is used, which is designed to represent the entire input sequence:


$$\begin{array}{c} z=H_{L}^{\left[\text{CLS}\right]} \end{array}$$ (5)

where $z\in {\mathbb{R}}^{d}$ is the feature vector for the [CLS] token, representing the global emotion of the entire text.

After RoBERTa’s feature extraction process, the social media text $T=[t_{1},t_{2},\ldots ,t_{n}]$ is mapped to a global emotion feature vector *z*:


$$\begin{array}{c} z=\text{RoBERTa}(T)=\text{LayerNorm}\left(H_{L}^{\left[\text{CLS}\right]}\right) \end{array}$$ (6)

where $H_{L}^{\left[\text{CLS}\right]}$ is the output of the final layer’s [CLS] token, normalized to produce the final global feature vector *z*, which is then passed to the subsequent Graph Neural Network (GNN) module for modeling emotion propagation and interaction among users.

Through this multi-level feature extraction process, Emotion-RGC Net is able to capture fine-grained emotion features from text. In the context of social media, this feature extraction lays a solid foundation for the subsequent stages of emotion propagation modeling and emotion classification.

### User relationship modeling module: GNN

In the Emotion-RGC Net model, the Graph Neural Network (GNN) is responsible for capturing user relationships and emotion propagation within social networks [32]. Since emotions are not only expressed by individuals but also propagated through interactions among users, relying solely on text feature extraction often fails to reflect the dynamic evolution of emotions [9]. To address this issue, GNN models the social relationships between users and incorporates the emotion propagation process into the emotion analysis framework, thereby enhancing the overall accuracy of emotion recognition. In this model, each user is represented as a node in a graph, with edges representing interactions between users (e.g., comments, likes, shares). By iteratively passing messages between nodes and updating their states, each user node learns not only from its own emotional features but also from the emotional states of its neighboring nodes, capturing the patterns of emotion propagation throughout the network. GNN models emotion propagation in the social network through the following iterative update formula:


$$\begin{array}{c} h_{i}^{\left(l+1\right)}=\sigma \left(W^{\left(l\right)}h_{i}^{\left(l\right)}+\text{AGG}\left(\left\{h_{j}^{\left(l\right)}:j\in N(i)\right\}\right)\right) \end{array}$$ (7)

**Fig 3:**
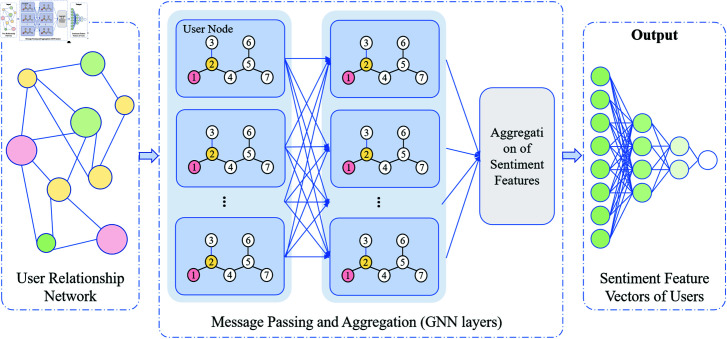
GNN Module Structure and Information Propagation Process. It includes a social network structure composed of nodes (users) and edges (interactions between users). Through multiple layers of message passing, nodes receive and aggregate emotional information from their neighboring nodes, gradually updating the emotional representation of each node.

As shown in Fig 3, GNN gradually updates the emotion representation of each node through a multi-layer message-passing mechanism. Initially, the feature vectors of the user nodes come from the text emotion features extracted by RoBERTa. GNN then aggregates information from neighboring nodes to update each node’s state. After multiple layers of propagation, the final emotion representation of each node incorporates both its own emotion features and those of its neighboring nodes. The GNN process consists of three steps: node initialization, message passing, and node updating, with the final output being feature vectors that encapsulate emotion propagation information.

The input to GNN is a graph structure *G* = ( *V* , *E* ) , where *V* represents the set of user nodes and *E* represents the interaction relationships between users. For each node $v_{i}\in V$, its initial feature vector is derived from the text features extracted by RoBERTa, $h_{i}^{0}\in {\mathbb{R}}^{d}$:


$$\begin{array}{c} h_{i}^{0}=\text{RoBERTa}(T_{i}) \end{array}$$ (8)

where $T_{i}$ is the text posted by user $v_{i}$, and $h_{i}^{0}$ is the emotion feature vector generated by RoBERTa, with *d* being the dimensionality of the feature vector.

The core of GNN is the message-passing mechanism, where information is propagated between nodes via their edges. In each round of message passing, a node $v_{i}$ receives information from its neighboring nodes $v_{j}\in N(i)$, and the features of the neighboring nodes are aggregated using an aggregation function AGG. The message-passing formula is as follows:


$$\begin{array}{c} m_{i}^{\left(l+1\right)}=\text{AGG}\left(\left\{h_{j}^{\left(l\right)}:j\in N(i)\right\}\right) \end{array}$$ (9)

where $m_{i}^{\left(l+1\right)}$ represents the message passed to node $v_{i}$ from its neighboring nodes in layer *l* + 1, and $h_{j}^{\left(l\right)}$ is the feature representation of neighboring node $v_{j}$ in layer *l*. The aggregation function AGG can be as simple as summation or averaging, or it can be more complex, such as max pooling or weighted summation.

After each round of message passing, the feature vector of each node is updated based on the aggregated information from its neighbors:


$$\begin{array}{c} h_{i}^{\left(l+1\right)}=\sigma \left(W^{\left(l\right)}h_{i}^{\left(l\right)}+m_{i}^{\left(l+1\right)}\right) \end{array}$$ (10)

where $W^{\left(l\right)}\in {\mathbb{R}}^{d\times d}$ is a learnable weight matrix, and *σ* is a non-linear activation function (such as ReLU). This process combines both the node’s own features and its neighbors’ information, allowing each node to capture the emotion propagation within the entire network.

After *L* layers of message passing and node updating, the final feature vector $h_{i}^{L}$ for each node contains the emotional features of both the node itself and its neighbors. The output of the GNN is the feature matrix $H_{G}\in {\mathbb{R}}^{|V|\times d}$, representing the emotional states of all nodes in the social network, where  | *V* |  is the number of nodes and *d* is the dimension of each node’s feature vector.


$$\begin{array}{c} H_{G}=\left[h_{1}^{L},h_{2}^{L},\ldots ,h_{\left|V\right|}^{L}\right] \end{array}$$ (11)

These feature vectors will serve as input to the next step in the pipeline, the Conditional Random Fields (CRF) module, for global optimization of emotion classification.

During the integration process, the embedding vector output by RoBERTa is converted into the input node features of GNN, and the relationship between nodes is further mined through the graph convolution layer of GNN, and information transmission and aggregation between nodes are performed with the support of the global graph structure. Through this integration strategy, we can effectively combine the semantic information of text and the spatial relationship of graph structure to improve the performance of the model in complex tasks.

### Post-processing module: CRF

Conditional Random Fields (CRF) are a widely used probabilistic model for sequence labeling tasks, particularly suited to handling problems with interdependent labels. CRF models the input features and the output label sequence jointly, considering the dependencies between labels to maximize the global probability of the label sequence [33]. In emotion classification tasks, CRF is particularly useful for capturing transitions between emotional states. For instance, a user’s emotional state across multiple social media posts is often continuous, or the emotional states of different users may become correlated through interactions [10].

In the Emotion-RGC Net model, the CRF module plays a key role in post-processing. While the GNN generates feature vectors for each user node, incorporating both their own emotional features and those of their neighbors, emotion classification is not simply an isolated task. Emotional states often exhibit continuity over time or correlations across user relationships. By modeling the dependencies between emotional states, CRF performs a global optimization on the emotion features output by the GNN, thus improving the accuracy of the final emotion classification.

**Fig 4:**
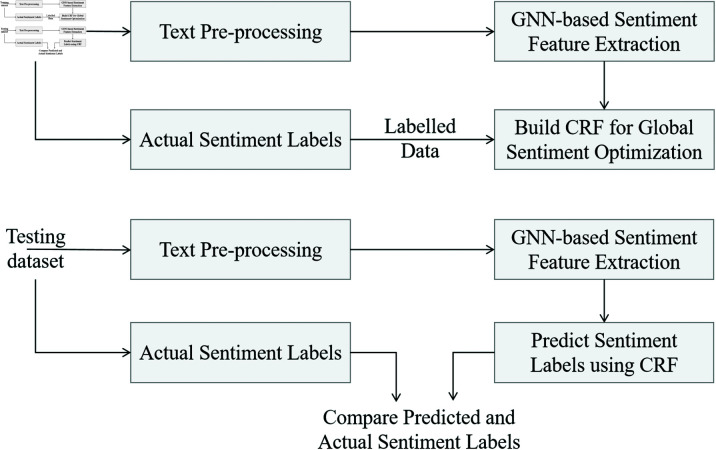
Structure and optimization process of the CRF module. CRF receives the node emotion features from GNN, calculates the matching score of each node’s emotion label using a feature function *ψ*, and models the dependencies between neighboring nodes’ labels using a label transition function *ϕ*. The Viterbi algorithm is then employed to globally decode the optimal emotion label sequence by maximizing the conditional probability.

As shown in Fig 4, CRF takes the feature vectors output by the GNN, $H_{G}=[h_{1}^{L},h_{2}^{L},\ldots ,h_{\left|V\right|}^{L}]$, as input and optimizes the global emotion classification by considering the dependencies between emotion labels. Each node’s feature vector $h_{i}^{L}$ corresponds to the predicted emotional state of user $v_{i}$. CRF not only considers the features of individual nodes but also introduces transition probabilities between labels to ensure coherence in the global emotion classification results.

In this model, CRF optimizes the emotion features output by GNN by modeling the joint probability of the emotion label sequence. Given the feature vectors $H_{G}$ from GNN, the goal of CRF is to predict the emotion labels $Y=[y_{1},y_{2},\ldots ,y_{\left|V\right|}]$ by maximizing the conditional probability $P(Y|H_{G})$, where $y_{i}$ is the emotion label of node $v_{i}$.

The conditional probability model for CRF is defined as:


$$\begin{array}{c} P(Y|H_{G})=\frac{1}{Z(H_{G})}\exp\left(\overset{\left|V\right|}{\underset{i=1}{\sum }}\psi (y_{i},h_{i}^{L})+\overset{|V|-1}{\underset{i=1}{\sum }}\phi (y_{i},y_{i+1})\right) \end{array}$$ (12)

where $\psi (y_{i},h_{i}^{L})$ is the feature function for node $v_{i}$, representing the compatibility between node $v_{i}$’s emotion label $y_{i}$ and its feature vector $h_{i}^{L}$. $\phi (y_{i},y_{i+1})$ is the transition function, modeling the dependencies between neighboring emotion labels $y_{i}$ and $y_{i+1}$, capturing the transition patterns of emotional states. $Z(H_{G})$ is the normalization factor that ensures the sum of the probability distribution equals 1:


$$\begin{array}{c} Z(H_{G})=\underset{Y^{\prime }}{\sum }\exp\left(\overset{\left|V\right|}{\underset{i=1}{\sum }}\psi (y_{i}^{\prime },h_{i}^{L})+\overset{|V|-1}{\underset{i=1}{\sum }}\phi (y_{i}^{\prime },y_{i+1}^{\prime })\right) \end{array}$$ (13)

The feature function $\psi (y_{i},h_{i}^{L})$ represents how well the emotion label $y_{i}$ matches the feature vector $h_{i}^{L}$ for node $v_{i}$, expressed through a linear model as:


$$\begin{array}{c} \psi (y_{i},h_{i}^{L})=W_{y}^{T}h_{i}^{L}+b_{y} \end{array}$$ (14)

where $W_{y}$ is the weight vector for emotion label *y*, and $b_{y}$ is a bias term. This function allows CRF to assign a preliminary probability to each node’s emotion label based on its feature vector.

The transition function $\phi (y_{i},y_{i+1})$ models the dependencies between consecutive emotion labels, ensuring that the emotional states of neighboring nodes do not change abruptly. It is defined as:


$$\begin{array}{c} \phi (y_{i},y_{i+1})=T_{y_{i},y_{i+1}} \end{array}$$ (15)

where $T_{y_{i},y_{i+1}}$ is the transition weight matrix representing the transition from emotion label $y_{i}$ to $y_{i+1}$. This matrix can be learned from historical data by capturing the transition patterns between labels.

CRF finds the optimal emotion label sequence *Y* by maximizing the conditional probability $P(Y|H_{G})$. Since directly computing the conditional probability for all possible label sequences is computationally expensive, the Viterbi algorithm is employed to efficiently find the most likely label sequence globally, ensuring the consistency and coherence of the final classification results:


Y∗= arg ⁡ max ⁡ YP(Y|HG) (16)

By introducing the transition function between labels and the feature matching function, CRF ensures that the emotion classification results are consistent both temporally and within the relational network. This global optimization process allows emotion classification to account for not only individual features but also the dynamic changes of emotions in context, significantly improving classification accuracy. The CRF module provides global optimization for the final output of Emotion-RGC Net, combining the local emotion features generated by the GNN with the coherence of the global emotion sequence, thus enhancing the model’s classification capability in social media emotion recognition.

## Experiment

### Datasets

To evaluate the effectiveness of Emotion-RGC Net in social media emotion recognition tasks, we conducted experiments using two widely utilized sentiment analysis datasets: the Sentiment140 Dataset and the Emotion Dataset. These datasets encompass a rich variety of social media content, offering diversity and representativeness, which effectively assess the model’s performance across different scenarios.

Sentiment140 is a well-established social media dataset in the sentiment analysis domain, primarily based on Twitter. It contains over 1.6 million tweets labeled with sentiment categories: positive, negative, and neutral. In addition to the tweet text, the dataset includes information about interactions between users, providing a foundation for modeling emotion propagation [34]. The tweets are typically short, highly informal, and feature a significant amount of emoticons, abbreviations, slang, and typos. Moreover, the subtlety and ambiguity in emotional expressions make emotion recognition particularly challenging. The Sentiment140 dataset is particularly suitable for testing the RoBERTa module of Emotion-RGC Net, specifically its capability to extract features from complex language structures, and for evaluating GNN’s performance in capturing user relationships and modeling emotion propagation.

The Emotion Dataset, in contrast, focuses on fine-grained multi-class emotion classification. Unlike Sentiment140, this dataset provides a richer set of emotion labels, including joy, anger, sadness, fear, surprise, disgust, and neutral. The data comes from social media comments, blogs, and conversation data, covering a wide range of emotional expressions and contexts [35]. This dataset offers an excellent opportunity to assess the CRF module in Emotion-RGC Net, particularly in its ability to enforce global consistency in emotion label optimization. Additionally, the dataset includes user interaction information, which can be used to further explore the paths and evolution of emotion propagation.

The Sentiment140 and Emotion datasets cover both coarse-grained and fine-grained emotion analysis tasks. The former focuses on basic sentiment classification, while the latter introduces more complex emotion categories. This diversity ensures that the experimental results span from fundamental to advanced emotion classification scenarios. Furthermore, both datasets are based on real-world social media content, providing high representativeness. The tweets, comments, and other content reflect the diverse forms of emotional expression prevalent on social media, enhancing the practical applicability of the experimental outcomes.

The Sentiment140 and Emotion datasets provide a comprehensive foundation for evaluating the performance of Emotion-RGC Net. They not only allow us to test RoBERTa’s ability to extract textual features but also validate GNN’s effectiveness in capturing user relationships and emotion propagation, as well as assess the contribution of the CRF module in optimizing global emotion classification.

### Experimental details

In our experiments, we conducted detailed parameter tuning for each module of Emotion-RGC Net to ensure the model effectively handles social media emotion recognition tasks. Additionally, multiple evaluation metrics were employed to measure the model’s performance, ensuring its competitiveness across various dimensions. The parameters of RoBERTa, GNN, and CRF modules were carefully adjusted to optimize the model’s performance on different datasets. The specific parameter settings for each module are presented in Table 1:

**Table 1:** Parameter Settings for Emotion-RGC Net.

Module	Parameter	Setting
RoBERTa	Pre-trained weights	RoBERTa-base
	Learning rate	2e-5
	Batch size	32
	Optimizer	Adam
	Dropout rate	0.1
GNN	Number of GCN layers	2
	Hidden dimensions per layer	64
	Learning rate	1e-3
	Aggregation method	Mean aggregation
	Regularization	L2 regularization
CRF	Label transition matrix initialization	Random initialization
	Optimization algorithm	Viterbi decoding
	Loss function	Maximum Likelihood Estimation (MLE)
Training Settings	Total epochs	10
	Loss function	Cross-entropy loss
	Optimizer	AdamW
	Learning rate decay	Step-wise learning rate decay

To comprehensively evaluate the performance of Emotion-RGC Net in emotion recognition tasks, we selected four key evaluation metrics. Accuracy measures the overall performance of the model by indicating the ratio of correctly classified samples to the total number of samples. Recall represents the model’s ability to identify actual positive samples. F1-score is the harmonic mean of precision and recall, balancing the model’s performance between these two aspects. AUC (Area Under the Curve) evaluates the model’s classification performance across various thresholds, with values ranging from 0 to 1, where a higher value indicates better classification performance.


$$\begin{array}{c} \text{Accuracy}=\frac{\text{TP}+\text{TN}}{\text{TP}+\text{TN}+\text{FP}+\text{FN}} \end{array}$$ (17)

$$\begin{array}{c} \text{Recall}=\frac{\text{TP}}{\text{TP}+\text{FN}} \end{array}$$ (18)

$$\begin{array}{c} \text{F1-score}=2\times \frac{\text{Precision}\times \text{Recall}}{\text{Precision}+\text{Recall}} \end{array}$$ (19)

These metrics provide a comprehensive evaluation of the model’s performance from different perspectives, ensuring that Emotion-RGC Net not only performs well in terms of accuracy but also effectively captures the true emotional categories. This is especially important when handling complex social media data, where robustness and generalization are critical.

In terms of data division, Sentiment140 Dataset contains over 1.6 million tweets, and after random shuﬄing, we divided them into the training and test set according to a ratio of 80% and 20%. For Emotion Dataset, this dataset contains about 100,000 text data of multi-category emotion labels, and we divided the training set by 70% , 15% and 15%, to ensure a balanced distribution of emotion categories in each dataset and avoid too many or too few samples in a certain category.

**Fig 5:**
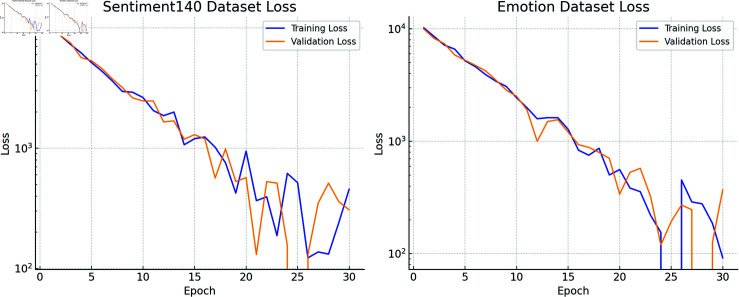
Training and validation loss curves on the Sentiment140 and Emotion datasets.

In addition, all texts are standardized and cleaned, removing HTML tags, URs and special characters, and using RoBERTa’s tokenizer to transform each text into a fixed-length word vector representation (length 256), truncated or populated if necessary to meet the input requirements of the model. For Sentiment140 Dataset, the number of samples of each category is relatively balanced by oversampling technology. For Emotion Dataset, due to the inclusion of seven emotion categories and uneven distribution of categories, the category samples were balanced by oversampling techniques to improve the diversity of the training set and the generalization ability of the model.

### Results analysis

From the loss curves (Fig 5), it can be observed that both the training loss and validation loss on the two datasets exhibit a significant downward trend as the training epochs increase, indicating that the model converges rapidly within a few training cycles. On the Sentiment140 dataset, the loss value drops sharply from an initially high value (around $10^{4}$) within the first 10 epochs, reflecting that Emotion-RGC Net effectively learns the emotional features of this dataset. A similar trend is observed on the Emotion dataset, where the initial loss is relatively high but quickly decreases within the first few epochs and gradually converges. This fast convergence can be attributed to the RoBERTa pre-trained model and the GNN module in the design. RoBERTa’s strong capability in extracting textual features allows it to capture key emotion-related information early on, accelerating the learning process. The GNN further captures the emotional propagation between users, helping the model quickly gain global insight during the early stages of training, which facilitates overall convergence.

On the Sentiment140 dataset, the validation loss closely mirrors the training loss, with only a small gap between the two, indicating that Emotion-RGC Net demonstrates good generalization on this dataset. In contrast, on the Emotion dataset, while the overall trend is similar, the validation loss shows greater fluctuation, particularly in the later stages of training (between 20 and 30 epochs), where a temporary increase in validation loss is observed. This suggests that the multi-emotion nature of the Emotion dataset poses greater challenges for the model, making it harder to generalize effectively when handling more complex emotion classification tasks. This phenomenon highlights that while Emotion-RGC Net performs stably in fine-grained emotion classification tasks (such as those in the Emotion dataset), its generalization capability on a broader range of emotion categories still requires further optimization. It also reflects that the model design of Emotion-RGC Net has a clear advantage in handling simpler emotion classification tasks, but improvements are needed when addressing more complex multi-class emotion classification.

From the loss curves, we can conclude that Emotion-RGC Net demonstrates good convergence and robustness on both datasets. The model’s training and validation losses remain stable, particularly on the Sentiment140 dataset, validating its effectiveness in coarse-grained emotion classification tasks. However, the greater fluctuation in validation loss on the Emotion dataset indicates that multi-class emotion classification presents a higher challenge for the model. Future work could focus on further optimizing the model structure and data processing methods to enhance performance in fine-grained emotion analysis.

**Fig 6:**
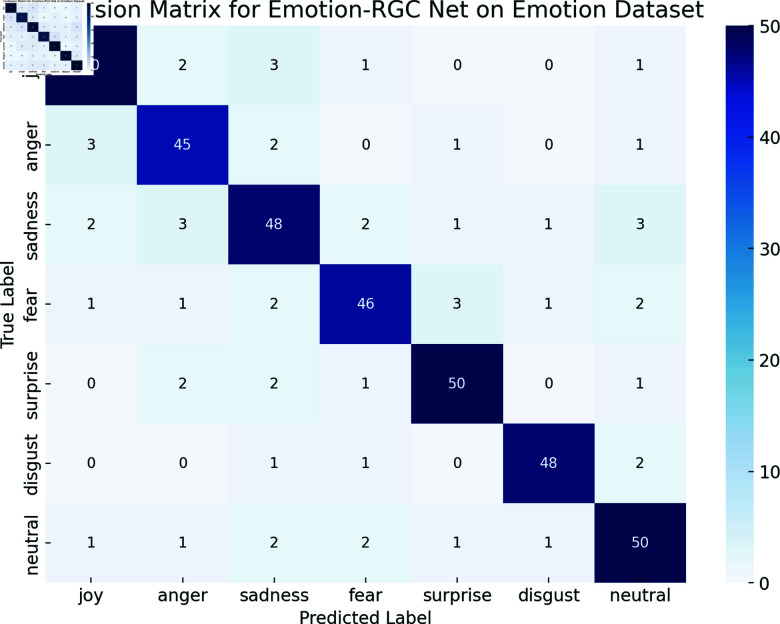
Confusion Matrix for the Emotion-RGC Net Model on the Emotion Dataset.

The Emotion dataset contains seven emotion categories, and compared with the three types of emotion labels in the Sentiment140 dataset, the task is undoubtedly more complex for fine-grained emotion classification in the Emotion dataset. We observed that the validation loss curve of the model showed large fluctuations on the Emotion dataset, especially in the late training period. While observing the results of the confusion matrix (Fig 6), we found that the classification accuracy of the "anger" and "fear" emotion categories was lower, while the classification performance of "happy" and "sadness" was more stable. This phenomenon indicates that there is some crossover and confusion between the emotion categories of the Emotion dataset, especially in the fine-grained emotion classification task, where the context of some emotion categories is similar, causing the model to accurately distinguish between these categories.

To further understand this phenomenon, we also performed detailed statistics on the accuracy, recall, and F1-score for each emotion category. The results showed that some categories in the Emotion dataset (such as "anger" and "fear") caused the model to perform poorly in the recognition of such emotions due to the ambiguity and polysemy of emotional expression. However, other categories such as "happy" and "sad" were more obvious due to the characteristics of emotional expression. Emotion-RGC Net shows strong ability in handling fine-grained emotion classification tasks, but also exposes limitations in multi-category emotion classification, especially when there is a large similarity or crossover between emotion categories.

As shown in the results of Table 2, we compared the performance of the Emotion-RGC Net model with several commonly used sentiment analysis models. Emotion-RGC Net demonstrated outstanding performance across various metrics on both the Sentiment140 and Emotion datasets, particularly excelling in key metrics such as accuracy, recall, F1-score, and AUC. On the Sentiment140 dataset, Emotion-RGC Net achieved an accuracy of 89.70%, and on the Emotion dataset, it reached 88.50%, significantly outperforming other models.

**Table 2:** Performance Comparison of Emotion-RGC Net and Other Benchmark Models on Sentiment140 and Emotion Datasets.

Models	Sentiment140 Dataset				Emotion Dataset			
	Accuracy	Recall	F1-score	AUC	Accuracy	Recall	F1-score	AUC
Naive Bayes [36]	76.57	77	77	0.85	76.10	77	76	0.83
Logistic Regression [37]	78.01	78	78	0.87	77.50	78	77	0.84
Decision Tree [38]	62.34	69	59	0.65	63.10	68	59	0.61
KNN [39]	60.39	66	60	0.63	60.50	66	60	0.64
AdaBoost [40]	69.94	71	70	0.77	70.20	71	69	0.75
GRU [41]	78.96	78	78	0.88	78.80	78	78	0.86
LSTM [6]	79.10	79	79	0.89	78.90	79	78	0.88
BiLSTM [42]	78.53	78	78	0.88	78.60	78	78	0.86
CNN-LSTM [5]	77.53	77	77	0.86	77.60	77	77	0.84
CNN-BiLSTM [43]	77.58	77	77	0.86	77.60	77	77	0.85
**Emotion-RGC Net**	**89.70**	**90**	**90**	**0.95**	**88.50**	**89**	**89**	**0.93**

Compared to traditional machine learning models such as Naive Bayes, Logistic Regression, and Decision Tree, Emotion-RGC Net showed substantial improvements. Specifically, on the Sentiment140 dataset, the accuracy of traditional models like Naive Bayes was 76.57%, and Logistic Regression reached 78.01%, whereas Emotion-RGC Net achieved 89.70%. This highlights the clear advantage deep learning models have in handling large-scale social media data, especially in capturing emotional context and complex relationships. Traditional models rely heavily on manual feature engineering, while Emotion-RGC Net leverages RoBERTa to automatically extract emotional features from text and uses GNN to model interactions between users, combined with CRF for global optimization. These results indicate that the design of Emotion-RGC Net significantly enhances both the accuracy and robustness of emotion classification.

However, despite RoBERTa’s excellent performance in sentiment feature extraction, we noticed that it may have some limitations when dealing with complex sentiment categories. Although RoBERTa can capture rich sentiment information, it may encounter the problem of "missing context" or "insufficient background information" when faced with the diversity and fine-grained distinction of sentiment categories. For example, when sentiment expression depends on deeper background knowledge or context dependence, the language model relying solely on RoBERTa may not be able to fully understand and distinguish these complex emotions. In our experiments, Emotion-RGC Net effectively utilizes the social relationship network between users by combining RoBERTa and GNN, thereby making up for the possible shortcomings of RoBERTa in dealing with subtle differences in sentiment categories. Specifically, GNN can capture the potential connections between different sentiment categories by modeling the interactive relationship between users, thereby improving the classification accuracy.

In comparison with other deep learning models, such as LSTM, GRU, and CNN-LSTM, Emotion-RGC Net still maintains a clear advantage. On the Sentiment140 dataset, LSTM achieved an accuracy of 79.10%, while Emotion-RGC Net reached 89.70%. Similarly, on the Emotion dataset, LSTM achieved 78.90%, whereas Emotion-RGC Net achieved 88.50%, showing a significant performance gap. Although models like LSTM and GRU are effective at handling sequential data and have shown promising results in sentiment analysis tasks, they are limited in capturing long-range dependencies and processing large-scale social media data. Emotion-RGC Net, by combining the strengths of RoBERTa and GNN, not only captures emotional features from individual texts but also utilizes the relational network between users to model emotion propagation, significantly boosting its classification ability.

Additionally, Emotion-RGC Net performed exceptionally well on both F1-score and AUC metrics. F1-score is the harmonic mean of precision and recall, and it provides a more balanced measure of a model’s performance, especially on imbalanced data. On the Sentiment140 dataset, Emotion-RGC Net achieved an F1-score of 90, and on the Emotion dataset, it reached 89, both far exceeding other models. This indicates that Emotion-RGC Net not only captures more positive samples but also avoids excessive false positives. In terms of AUC (Area Under the Curve), Emotion-RGC Net scored 0.95 on Sentiment140 and 0.93 on the Emotion dataset, while other models typically had AUCs around 0.85. This further confirms that Emotion-RGC Net excels in distinguishing between positive and negative classes across different thresholds, which is crucial for accurate classification in sentiment analysis tasks.

From the above results, we can see that Emotion-RGC Net not only performs well in traditional sentiment analysis tasks, but also shows strong advantages when facing the fine-grained problem of sentiment classification. The superiority of RoBERTa in sentiment feature extraction combined with the advantage of GNN in capturing relationships between users greatly improves the overall performance of the model and makes up for the limitations of RoBERTa in dealing with complex sentiment categories.

Table 3 presents the results of the ablation study, which demonstrates that each module of the Emotion-RGC Net is critical for improving overall performance.

**Table 3:** Results of Emotion-RGC Net ablation experiment on Sentiment140 and Emotion datasets.

Models	Sentiment140 Dataset			Emotion Dataset		
	Accuracy	Recall	F1-score	Accuracy	Recall	F1-score
Full Model (Emotion-RGC Net)	89.70	90	90	88.50	89	89
Without CRF	86.45	87	87	85.20	86	86
Without GNN	84.30	85	85	82.40	83	83
Without RoBERTa	79.10	80	79	78.90	80	79
Without GNN and CRF	78.00	78	77	76.80	78	77
Without RoBERTa and CRF	75.50	76	76	74.20	75	75

When any module is removed, significant drops in accuracy, recall, and F1-score are observed on both the Sentiment140 and Emotion datasets. Particularly, the removal of the RoBERTa and CRF modules results in substantial declines in performance. After removing RoBERTa, the accuracy on the Sentiment140 dataset drops from 89.70% to 79.10%, highlighting RoBERTa’s pivotal role in extracting emotional features from text. Similarly, removing the CRF module significantly affects the model’s performance, especially in terms of consistency in emotion classification (F1-score). CRF’s global optimization of emotion labels ensures coherent classification, and when this module is removed, the F1-score decreases by more than 3 percentage points.Moreover, the GNN module plays a crucial role in capturing the propagation of emotions between users. When the GNN module is removed, the model’s performance also experiences a substantial decline, indicating that the information between users is essential for optimizing emotion classification. These results collectively suggest that the design of Emotion-RGC Net is both rational and effective, with the collaboration of each module significantly enhancing the model’s performance in emotion recognition tasks.

**Fig 7:**
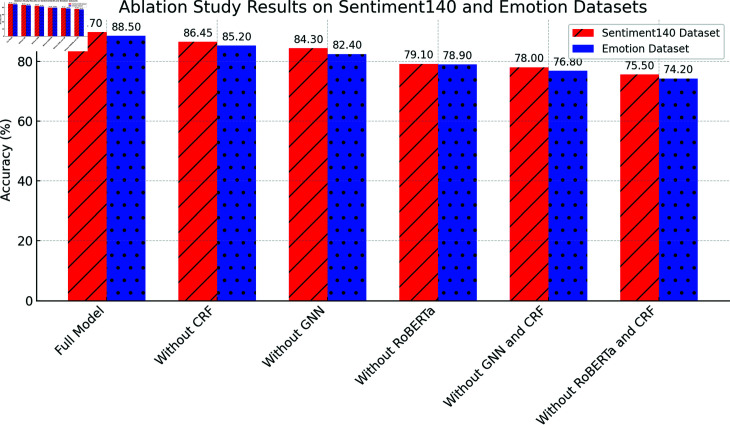
Ablation Study: Accuracy On Sentiment140 And Emotion Datasets.

**Fig 8:**
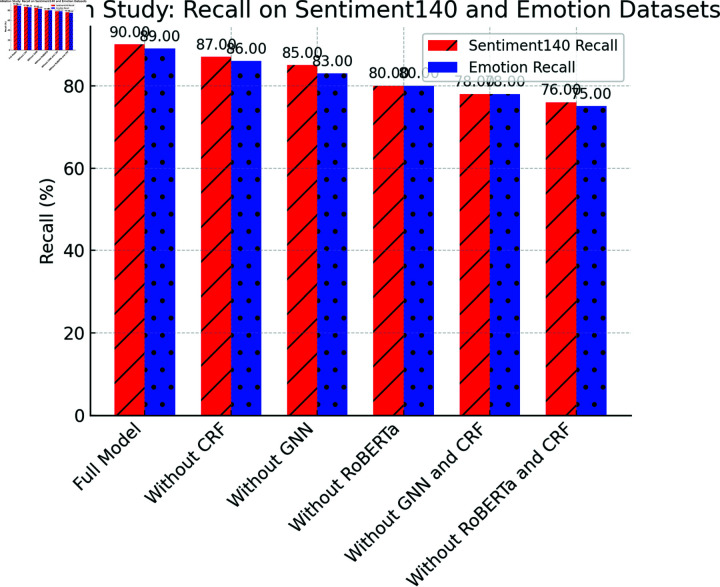
Ablation Study: Recall On Sentiment140 And Emotion Datasets.

**Fig 9:**
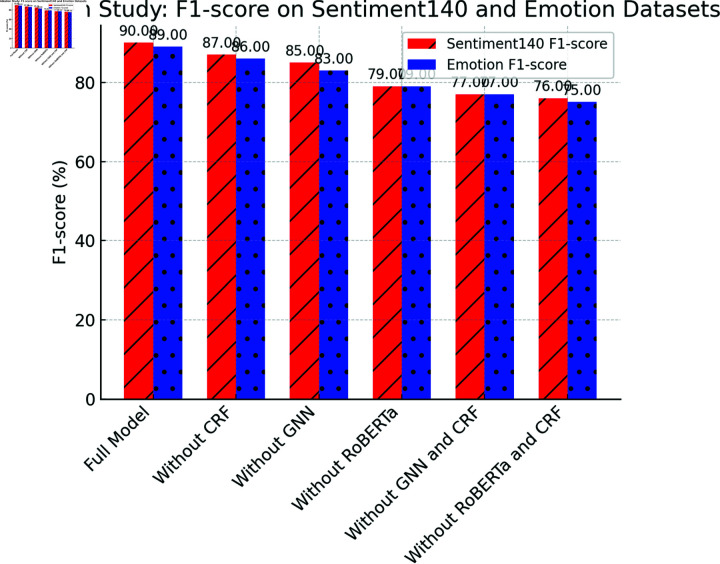
Ablation Study: F1-Score On Sentiment140 And Emotion Datasets.

Figs 7, 8 and 9 illustrates the visualization of the ablation study results for the Emotion-RGC Net model on both the Sentiment140 and Emotion datasets.

Each module in the Emotion-RGC Net model plays an indispensable role in emotion recognition tasks. Especially when dealing with complex social media sentiment analysis tasks, the collaborative work of each module significantly improves the overall performance of the model. RoBERTa, as an emotional feature extraction module, plays a crucial role in extracting emotional information from text. After removing RoBERTa, the accuracy of the model dropped significantly, especially on the Sentiment140 data set, where the accuracy dropped from 89.70% to 79.10%. This shows that RoBERTa’s powerful text understanding capabilities play a key role in the model and can effectively capture emotion-related fine-grained features. Without RoBERTa, the model cannot extract enough emotional information from the text, resulting in significant performance degradation.

The removal of the CRF module also has a significant impact on model performance. CRF optimizes the consistency of emotion classification by modeling the global dependencies between emotion labels, especially in the temporal continuity and logical relationships between labels in emotion classification tasks. After removing CRF, the model’s F1-score dropped by more than 3 percentage points, indicating that after the relationship between emotional labels was ignored, more inconsistencies and errors appeared in the classification results. This result highlights the global optimization role of CRF in emotion classification tasks, especially when dealing with emotion labels with dependencies and temporal sequence.

In addition, the removal of the GNN module also has a greater impact on model performance. The core advantage of GNN is to capture the emotional communication relationship between users through the graph structure, which is crucial for social media sentiment analysis, because emotions are often not only expressed by individuals, but also spread through interactions in social networks. After removing the GNN module, the performance of the model dropped significantly, especially in terms of capturing the emotional propagation and dependence between users, and the accuracy and generalization ability of the model were significantly reduced. This result further proves the important role of GNN in emotional propagation modeling. Especially in large-scale social network data, the impact of interactive relationships between users on emotional analysis cannot be ignored.

The module design of Emotion-RGC Net is well thought out, and the collaborative work of each module significantly enhances the model’s performance in complex emotion classification tasks. RoBERTa provides the model with powerful text feature extraction capabilities, GNN provides additional contextual information by modeling the emotional communication relationship between users, and CRF further optimizes the global consistency of the classification results on this basis.

## Conclusion

This paper proposes a novel emotion recognition model Emotion-RGC Net, combining three modules: RoBERTa, graph Neural Network (GNN) and conditional random field (CRF), aiming to improve the accuracy and robustness of emotion analysis in social media. The large-scale experimental results on Sentiment140 and Emotion datasets show that Emotion-RGC Net performs well in all metrics, especially in core metrics such as accuracy, recall and F1-score, and significantly outperforms traditional machine learning models and other deep learning models. RoBERTa The pre-training model plays an important role in processing the unstructured text of social media and extracting emotional features. By modeling the interaction between users, GNN captures the transmission and influence process of emotion in social networks, and effectively improves the accuracy of emotion recognition. CRF further enhances the global consistency and coherence of emotion classification results by modeling the dependence of emotion labels.

Despite the excellent experimental results of Emotion-RGC Net, the model still has some limitations, especially in terms of computational complexity and data dependency. First, the model relies on pre-trained RoBERTa and large-scale annotated data, and may face performance degradation when dealing with low-resource languages or data-scarce sentiment analysis scenarios. Second, the computational complexity of the GNN module is high, which may lead to a lot of time and resource consumption, especially when dealing with large-scale social networks. On the other hand, although the global optimization mechanism of CRF can improve the consistency of emotion classification, it may have limited effect when dealing with extreme emotions or atypical emotional expressions.

Future research will focus on enhancing the model’s performance in small-sample or unlabeled data scenarios through data augmentation or unsupervised learning techniques. Additionally, we aim to incorporate more emotional dimensions or fine-grained emotional categories to address more complex sentiment analysis challenges. As social media content becomes increasingly diverse and real-time, the dynamic temporal changes of emotions are also becoming an important issue. We plan to explore how to integrate temporal dynamics to capture the evolving patterns of emotions in social networks, thus improving the applicability and robustness of Emotion-RGC Net.

Emotion-RGC Net not only holds significant technical value but also demonstrates profound societal impact, particularly in the areas of online emotional support and mental health intervention. By analyzing users’ emotions in real time on social media, Emotion-RGC Net can provide timely and effective emotional support for online mental health interventions, helping to identify users experiencing emotional distress and providing relevant organizations with scientifically backed intervention data. This is of critical importance in addressing the growing mental health challenges in modern society. Additionally, Emotion-RGC Net can play a key role in social sentiment monitoring, where it can track public emotion fluctuations, offering governments and businesses early warnings on public sentiment and social dynamics. This can assist decision-makers in optimizing public policies and business strategies.

However, with the widespread application of sentiment recognition technology, privacy protection and data ethics issues are becoming increasingly prominent challenges. Sentiment analysis models rely on users’ personal data for training, which may involve the collection and use of sensitive information. Therefore, ensuring user privacy, data security, and adhering to ethical standards during model development and deployment are critical concerns. To address these, we recommend implementing strict privacy protection measures when using Emotion-RGC Net, such as data encryption and anonymization techniques. Additionally, transparency regarding data sources and usage purposes should be strengthened to ensure that user privacy is safeguarded and personal rights are not violated. We believe that by continuously optimizing the model and reinforcing ethical oversight, Emotion-RGC Net will better serve society and contribute to the healthy development of sentiment analysis technology.
